# Rotational Coupling in Methyl-Tunneling Electron Spin Echo Envelope Modulation

**DOI:** 10.1007/s00723-021-01375-6

**Published:** 2021-07-14

**Authors:** Gunnar Jeschke

**Affiliations:** Department of Chemistry and Applied Biosciences, ETH Zürich, Vladimir-Prelog-Weg 2, 8093 Zürich, Switzerland

## Abstract

Coherence between tunnel-split states of a methyl quantum rotor can be generated and observed in stimulated and spin-locked echo experiments, if hyperfine coupling of a nearby electron spin to the methyl protons breaks C$_3$ symmetry and is of the same order of magnitude as the tunnel splitting. Here, we consider the case of two methyl groups bound to the same sp$^{3}$-hybridized atom, which is important in the context of common nitroxide spin labels. For a simple form of the rotor-rotor coupling Hamiltonian, we provide an approach that allows for density operator computations of this system with 1152 quantum states with moderate computational effort. We find that, in the regime where the ratio between rotor-rotor coupling and rotational barrier is much smaller than unity, three-pulse ESEEM and hyperfine-decoupled ESEEM depend only on the tunnel splitting, but not on this ratio. This finding may simplify the treatment of tunnel-induced electron decoherence in systems where the methyl groups are bound to sp$^{3}$-hybridized atoms.

## Introduction

Methyl groups are ubiquitous in materials and biopolymers as well as in many organic solvents. At ambient temperature and above, they rotate fast on the timescale of hyperfine anisotropy of their protons. At temperatures around 80 K and slightly below, their rotation is still sufficiently fast to induce changes in the hyperfine field at a nearby electron spin that are observable in electron spin echo (ESE) experiments [1]. Rotation of matrix methyl groups [2] as well as of methyl groups belonging to a paramagnetic species [3] contributes to phase memory loss (decoherence) of electron spins, thus limiting resolution of ESE experiments. Surprisingly, the presence of methyl groups still enhances decoherence in a temperature range where their rotation is by orders of magnitude slower than the phase memory time. Therefore, and because of a correlation between phase memory time and the rotation barrier, it was hypothesized that methyl tunneling leads to electron spin decoherence in the temperature range from 40 K down to at least 11 K and probably also below [4]. Indeed, methyl group tunneling effects on EPR spectra were recognized as early as 1972 [5] and a 1998 theoretical treatment suggested electron spin echo envelope modulation (ESEEM) of the two-pulse echo due to methyl tunneling [6]. Recently, we observed magnetic-field independent three-pulse ESEEM in an Mn(II)-doped metal-organic framework containing dimethylammonium (DMA) cations [7] and assigned it to coherence between methyl group tunnel states [8]. The three-pulse ESEEM signature of tunneling is distinct from other ESEEM effects by the magnetic-field independence of, both, frequency and modulation depth. The spectra revealed more transition frequencies than expected, even after hyperfine decoupling. We tentatively assigned this effect to quantum-rotor coupling [9] between the two methyl groups of the DMA cation. Here we test this hypothesis by developing theory for the ESEEM effect due to two rotationally coupled methyl groups and by numerical simulations. We assume a simple model Hamiltonian for the rotor-rotor coupling and address the question whether the barrier height of a single rotor and rotor-rotor coupling can be separated by tunnel ESEEM. Such treatment is also of interest for understanding electron spin decoherence due to internal methyl groups of nitroxide spin labels at low temperatures [10], as common nitroxides contain two pairs of geminal methyl groups.

This paper is structured as follows. First, we derive the sequence of basis transformations and level orderings that allow us to formulate the spin Hamiltonian on a basis of localized states for the two methyl groups. This step involves transformation of the rotor-rotor coupling Hamiltonian to the localized basis. We discuss the effects of state mixing induced by this coupling Hamiltonian. Second, we demonstrate that, in situations of practical interest, EPR experiments are confined to a single ro-vibrational state. We discuss computational limitations to the accuracy of deriving the Hamiltonian as well as the excitation and detection operators for such ro-vibrational states. Third, we show that with approximate solutions for these operators in hands, it is feasible to predict the outcome of any pulse EPR experiment on such a system by density operator formalism. We illustrate this approach by computations for rotation barriers and coupling potentials that lead to tunnel frequencies in the ESEEM range. We conclude with a general assessment of the information that can and cannot be obtained by such experiments on the quantum-rotor system.

**Fig. 1:**
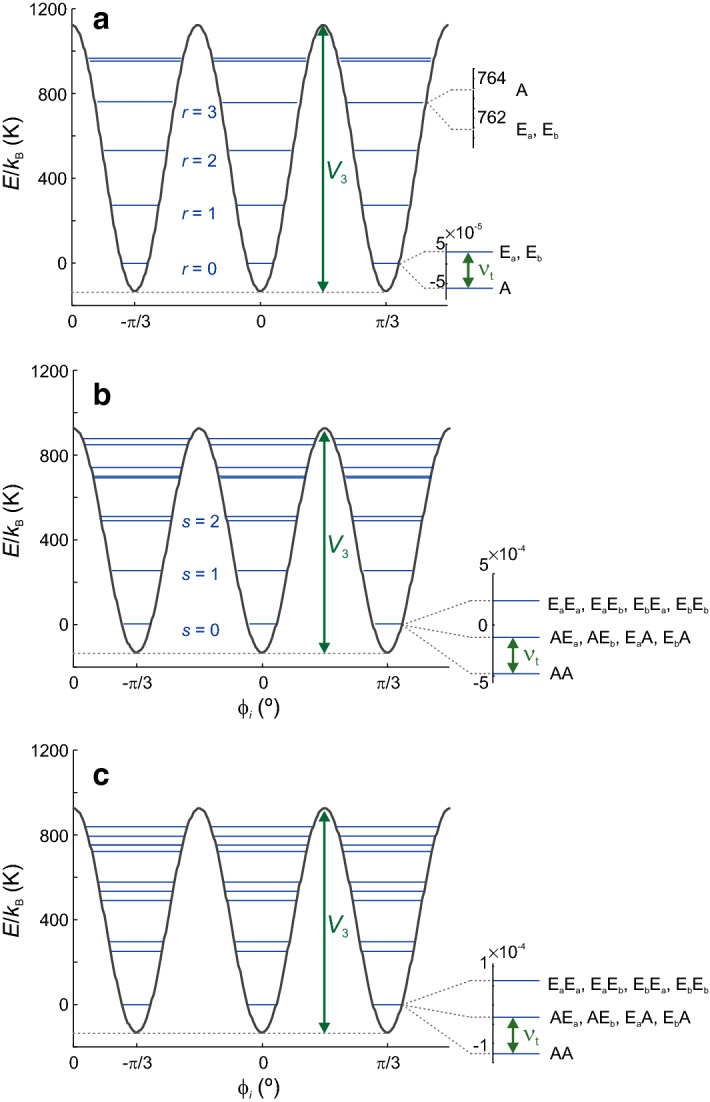
Energy level schemes for a single methyl rotor with rotation barrier $V_3 = 10.5$ kJ/mol corresponding to 1263 K (**a**), two uncoupled methyl rotors with $V_3 = 8.785$ kJ/mol corresponding to 1057 K (**b**), and two coupled rotors with $V_3 = 8.785$ kJ/mol and coupling $W_3 = -1$ kJ/mol (**c**). The ro-librational levels $r = 0, 1, \ldots$ are tunnel-split, as indicated on the right. For even *r*, the A level has lower energy than the E levels, for odd *r* it is the opposite. The principal tunnel splitting $\nu _t$ for $r=0$ is matched between the cases (**a**) and (**c**). Quantum number $s = r_1 + r_2$ is the sum of the ro-librational quantum numbers of the two rotors

## Single Methyl Rotor

We start with a reformulation of our previous treatment for a single rotor [8] that provides a better basis for advancing to the case of two coupled rotors. The Hamiltonian for a single quantum rotor is given by

1 $$\begin{aligned} {\mathcal {H}}_\mathrm {rot}\left( \phi \right) = -B \left( \frac{\partial ^2}{\partial \phi ^2} \right) + \frac{V_3}{2} \cos 3 \phi \ , \end{aligned}$$

where $B = \hbar /2I^2 = 0.655$ meV is the rotational constant of the methyl rotor, *I* is its moment of inertia, $\phi$ the rotational coordinate, and $V_3$ the rotation barrier. For a more convenient discussion of state populations, we express this Hamiltonian in units of K for numerical computations ($B = 7.6$ K). Note that our definition for $V_3$ differs by a factor of 1/2 from the one followed by Khazaei and Sebastiani [9] to associate this parameter with the activation barrier (Fig. 1) used in most literature on the topic. We convert the potential given in kJ/mol to temperature units by dividing it by the universal gas constant $R = 8.314 \ \mathrm {J} \ \mathrm {mol}^{-1} \ \mathrm {K}^{-1}$.

The Hamiltonian can be expressed with basis states for the free rotor ($V_3 = 0$), which have period $2 \pi$ with respect to $\phi$, leading to an infinite, but discrete set of basis states. Following earlier treatments, we truncate this set to a total of $2K+1$ states indexed by $-K, -K+1, \ldots K$, where we select a *K* so that $2K+1$ is a multiple of 3. An appropriate value of *K* can be found by checking convergence of the tunnel frequency and of the energies of the ro-librational states with increasing *K*. The finite-dimensional Hamiltonian can be expressed as

2 $$\begin{aligned} {\mathcal {H}}_\mathrm {rot} \approx B {\mathbb {C}} + \frac{V_3}{4} \left( {\mathbb {I}}_{+3} + {\mathbb {I}}_{-3} \right) \ . \end{aligned}$$

The matrices ${\mathbb {C}}$, ${\mathbb {I}}_{+3}$, and ${\mathbb {I}}_{-3}$ can be found in the Appendix of [9]. The solution of the time-independent Schrödinger equation for this Hamiltonian is a set of ro-librational states that we index by quantum number $r = 0 \ldots R-1$ ($R = (2K+1)/3$) and that have energies $\epsilon (r)$. Each ro-librational state has three tunnel substates. The matrix ${\mathbf {V}}$ of their eigenvectors ensures that ${\mathbf {V}}' {\mathcal {H}}_\mathrm {rot} {\mathbf {V}}$ is diagonal. We sort states in the order of increasing energy. Among the tunnel substates corresponding to a certain value of *r*, the states E$_\mathrm {a}$ and E$_\mathrm {b}$ are degenerate. The energy difference between this pair of levels and the A level is the tunnel splitting $\nu _{\mathrm {t},r}$, which increases with increasing *r*. For even *r*, the A state has lower energy than the two E states, whereas for odd *r* it has higher energy (Fig. 1a). For each ro-librational state, we can formulate a subspace Hamiltonian in the basis of localized rotational states

3 $$\begin{aligned} {\mathcal {H}}_{r}^\mathrm {loc} = \begin{bmatrix} \epsilon (r) &{} -\nu _{\mathrm {t},r}/3 &{} -\nu _{\mathrm {t},r}/3\\ -\nu _{\mathrm {t},r}/3 &{} \epsilon (r) &{} -\nu _{\mathrm {t},r}/3 \\ -\nu _{\mathrm {t},r}/3 &{} -\nu _{\mathrm {t},r}/3 &{} \epsilon (r) \end{bmatrix} \end{aligned}$$

We assign the states of this subspace Hamiltonian by a quantum number *q* that runs from 0 to 2.

In the following, we correct the eigenvectors of ${\mathcal {H}}_{r}^\mathrm {loc}$ that we misprinted in the Supplementary Material of [8]. In addition, for aesthetic reasons we choose a symmetrized representation for the two E states. Analytical diagonalization of ${\mathcal {H}}_{r}^\mathrm {loc}$ provides

4 $$\begin{array}{ll} {v_{{\text{A}}} = \frac{1}{{\sqrt 3 }}\left[ {1,1,1} \right]} \\ {v_{{{\text{E}}_{{\text{a}}} }} = \frac{1}{{\sqrt 3 }}\left[ {\left( {1 + \sqrt 3 } \right)/2,\left( {1 - \sqrt 3 } \right)/2, - 1} \right]} \\ {v_{{{\text{E}}_{{\text{b}}} }} = \frac{1}{{\sqrt 3 }}\left[ {\left( {1 - \sqrt 3 } \right)/2,\left( {1 + \sqrt 3 } \right)/2, - 1} \right]} \\ \end{array}$$

which we arrange in a matrix ${{\varvec{D}}}$, so that

5 $$\begin{aligned} {{\varvec{D}}}' {\mathcal {H}}_{r}^\mathrm {loc} {{\varvec{D}}} = \epsilon (r) + \begin{bmatrix} -2\nu _{\mathrm {t},r}/3 &{} 0 &{} 0\\ 0 &{} \nu _{\mathrm {t},r}/3 &{} 0 \\ 0 &{} 0 &{} \nu _{\mathrm {t},r}/3 \end{bmatrix} \end{aligned}$$

gives the sub-space Hamiltonian in the delocalized eigenbasis for even *r*. For odd *r*, we reverse the order of the eigenvectors. With the $(2K+1) \times (2K+1)$ matrix ${{\varvec{L}}} = {\mathbb {E}}_R \otimes {{\varvec{D}}}$ we can transform ${\mathcal {H}}_\mathrm {rot}^\mathrm {EB}$ from its eigenbasis to the basis of localized states by ${\mathcal {H}}_\mathrm {rot}^\mathrm {loc} = {{\varvec{L}}} {\mathcal {H}}_\mathrm {rot}^\mathrm {EB} {{\varvec{L}}}'$. Here, ${\mathbb {E}}_R$ is an $R \times R$ unit matrix. In the localized basis, we can construct the total Hamiltonian including interactions of a nearby electron spin and the three methyl protons as described in [8]. Here, we will construct the spin Hamiltonian for one electron spin and six protons only after extending the treatment to two uncoupled rotors.

**Fig. 2:**
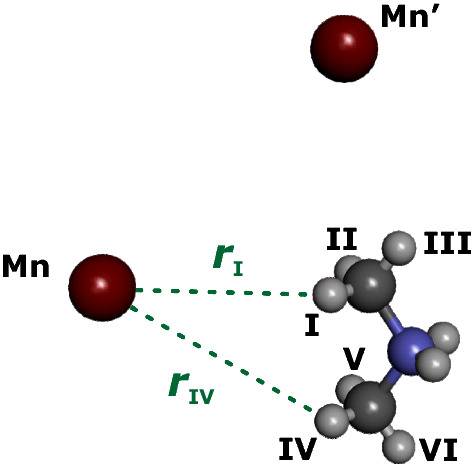
Example for the geometry of the two-rotor problem based on the crystal structure of [(CH$_3$)$_2$NH$_2$][Mn(HCOO)$_3$] [11]. The electron spin is localized on an Mn(II) ion, such as Mn or Mn’ (red). The two methyl groups are equivalent, except for the hyperfine interaction of protons I, II, III (methyl group 1) and IV, V, and VI (methyl group 2) with the electron spin. The hyperfine interaction breaks symmetry, since the vectors ${{\varvec{r}}}_k$ with $k =$ I, II, III, IV, V, VI in general all differ in length and orientation. For clarity, only ${{\varvec{r}}}_\mathrm {I}$ and ${{\varvec{r}}}_\mathrm {IV}$ for ion Mn are indicated (green), whereas formate ligands, and the remaining six Mn(II) ions are omitted (Color figure online)

## Two Uncoupled Methyl Rotors

We consider the case of two uncoupled methyl rotors with identical rotation barrier $V_3$. As an example, we picture the case of a DMA cation in the vicinity of an Mn(II) ion (Fig. 2), disregarding any rotor-rotor coupling for the moment. The quantum rotor Hamiltonian is now given by

6 $$\begin{aligned} {\mathcal {H}}_\mathrm {rot}\left( \phi _1 , \phi _2\right) = -B \left( \frac{\partial ^2}{\partial \phi _1^2} + \frac{\partial ^2}{\partial \phi _2^2} \right) + \frac{V_3}{2} \cos 3 \phi _1 + \frac{V_3}{2} \cos 3 \phi _2 \ . \end{aligned}$$

For the matrix representation of the truncated Hamiltonian, we have

7 $$\begin{aligned}&{\mathcal {H}}_\mathrm {rot} \approx \left[ B {\mathbb {C}} + \frac{V_3}{4} \left( {\mathbb {I}}_{+3} + {\mathbb {I}}_{-3} \right) \right] \otimes {\mathbb {E}}_{2K+1} \end{aligned}$$

8 $$\begin{aligned}&+ {\mathbb {E}}_{2K+1} \otimes \left[ B {\mathbb {C}} + \frac{V_3}{4} \left( {\mathbb {I}}_{+3} + {\mathbb {I}}_{-3} \right) \right] \ . \end{aligned}$$

The eigenstates of this truncated Hamiltonian are identified by a set of quantum numbers $\left( r_1, q_1, r_2, q_2 \right)$. Their energies are given by

9 $$\begin{aligned} \epsilon \left( r_1, q_1, r_2, q_2 \right) = \epsilon \left( r_1, q_1 \right) + \epsilon \left( r_2, q_2 \right) \end{aligned}$$

and the Hamiltonian in Eq. (8) is diagonalized by

10 $$\begin{aligned} {\mathcal {H}}_\mathrm {rot}^\mathrm {EB} = \left( {{\varvec{V}}} \otimes {{\varvec{V}}} \right) ' {\mathcal {H}}_\mathrm {rot} \left( {{\varvec{V}}} \otimes {{\varvec{V}}} \right) \ . \end{aligned}$$

This Hamiltonian of two uncoupled rotors in its eigenbasis is not energy-ordered. The ordering of states is defined by the outer product of the eigenvector matrix of the single-rotor problem ${{\varvec{V}}}$ with itself. To arrive at the analog of the tunnel subspace formulation in Eq. (5), we reorder the states as follows. We assign a total ro-librational quantum number $s = r_1 + r_2$. For small *s* and $V_3 \gg B$, the harmonic approximation $\cos \phi _i \approx 1 - \phi _i^2/2$ holds. Within this approximation, the ro-librational levels in the single-rotor problem are equidistant and the ro-librational energy contribution in the two-rotor problem is the same for all values of *s*. Therefore, we order the states by increasing *s*. Within a given *s*-subspace ($s \le R-1$), we order by $r_1 = 0 \ldots S$, corresponding to $r_2 = s, s-1 \ldots 0$. For a given pair $\left( r_1, r_2 \right)$, we order the 9 tunnel substates by $\left( q_1, q_2\right) = \left( 0, 0 \right) , \left( 0 , 1 \right) , \left( 0, 2 \right) , \left( 1 , 0 \right) \ldots$ The latter ordering ensures that the matrix ${\varvec{D}}(r_1) \otimes {\varvec{D}}(r_2)$ can be used for interconversion between the localized and delocalized subspace basis. Note that ${\varvec{D}}$ depends only on parity of $r_1$ and $r_2$. The index *k* for a set of quantum numbers $\left( r_1, q_1, r_2, q_2 \right)$ in the matrix representation of ${\mathcal {H}}_\mathrm {rot}^{EB}$ is given by

11 $$\begin{aligned} k\left( r_1, q_1, r_2, q_2 \right) = 9 R r_1 + 3 R q_1 + 3 r_2 + q_2 \ , \end{aligned}$$

assuming that we count states of ${\mathcal {H}}_\mathrm {rot}^\mathrm {EB}$ starting from $k = 0$. After reordering, the matrix ${\mathbf {L}}$ for interconversion between the delocalized and localized basis can be constructed by assigning each of the $R^2$ diagonal blocks of dimension $9 \times 9$ a parity pair even/even, even/odd, odd/even, or odd/odd, depending on parity of $r_1$ and $r_2$ and generating the appropriate block matrix ${\varvec{D}}(r_1) \otimes {\varvec{D}}(r_2)$.

We are now able to compute the analog of the rotor Hamiltonian in the localized basis (Eq. 3) for each $9 \times 9$ block corresponding to a ro-librational state ($r_1,r_2$) of two uncoupled methyl groups. This puts us in the position to construct the matrix representation of the Hamiltonian for two uncoupled methyl groups that are hyperfine coupled to a nearby electron spin.

**Table 1:** Assignment of secular hyperfine couplings $A_i$ for the nine localized rotor states $(\phi _1,\phi _2)$

$\phi _1$	$A_1$	$A_2$	$A_3$	$\phi _2$	$A_4$	$A_5$	$A_6$
$-\pi /3$	$A_\mathrm {I}$	$A_\mathrm {II}$	$A_\mathrm {III}$	$-\pi /3$	$A_\mathrm {IV}$	$A_\mathrm {V}$	$A_\mathrm {VI}$
$\pi /3$	$A_\mathrm {II}$	$A_\mathrm {III}$	$A_\mathrm {I}$	$\pi /3$	$A_\mathrm {V}$	$A_\mathrm {VI}$	$A_\mathrm {IV}$
$\pi$	$A_\mathrm {III}$	$A_\mathrm {I}$	$A_\mathrm {II}$	$\pi$	$A_\mathrm {VI}$	$A_\mathrm {IV}$	$A_\mathrm {V}$

Assignment of pseudo-secular hyperfine couplings $B_i$ is analogous

## Construction of the Spin Hamiltonian

In construction of the spin Hamiltonian, we restrict ourselves to the lowest ro-librational state $r=0$. This corresponds to a low-temperature approximation regarding ro-librational excitation, which is permitted if contributions from subspaces with $r>0$ to the spin echo signal are negligible. Our treatment thus applies at temperatures

12 $$\begin{aligned} T \ll \frac{E(r=1)-E(r=0)}{k_\mathrm {B}} \ . \end{aligned}$$

We note that for $T < 5[E(r=1)-E(r=0)]/k_\mathrm {B}$ less than one percent of the system is in subspaces with $r>0$. For instance, in the absence of rotor-rotor coupling ($W_3 = 0$, see Sect. 5) the approximation holds below 50 K for $V_3 \ge 10$ kJ/mol and below 12 K for $V_3 \ge 1$ kJ/mol. For the approximation to hold below 12 K in the presence of rotor-rotor coupling at $V_3 = 10$ kJ/mol, $W_3$ can be as large as 3.5 kJ/mol.

The spin system consists of one electron spin $S=1/2$ and six proton spins $I_{1} = I_{2} = I_{3} = I_{4} = I_{5} = I_{6} = 1/2$, where indices 1, 2, 3 refer to the first methyl group with rotor quantum numbers $r_1, q_1$ and indices 4, 5, 6 to the second methyl group with quantum numbers $r_2, q_2$. The hyperfine couplings $A_\mathrm {I}$, $A_\mathrm {II}$, $A_\mathrm {III}$, $A_\mathrm {IV}$, $A_\mathrm {V}$, and $A_\mathrm {VI}$ are associated with spatial proton positions denoted by Roman numerals (Fig. 2). The difference between the three localized rotor states of the first methyl group ($\phi _1 = -\pi /3, \pi /3, \pi$) lies in the assignment of the hyperfine couplings I, II, III to the protons 1, 2, 3 as described in [8]. The situation is analogous for the second methyl group. For a given localized state $(\phi _1,\phi _2)$, the spin Hamiltonian is

13 $$\begin{aligned} {\mathcal {H}}_\mathrm {spin}\left( \phi _1,\phi _2 \right)\,=\, & {} \omega _S S_z + \omega _I \left( I_{1z} + I_{2z} + I_{3z} + I_{4z} + I_{5z} + I_{6z} \right) \nonumber \\&+ A_1(\phi _1) S_z I_{1z} + A_2(\phi _1) S_z I_{2z} + A_3(\phi _1) S_z I_{3z} \nonumber \\&+ A_4(\phi _2) S_z I_{4z} + A_5(\phi _2) S_z I_{5z} + A_6(\phi _2) S_z I_{6z} \nonumber \\&+ B_1(\phi _1) S_z I_{1x} + B_2(\phi _1) S_z I_{2x} + B_3(\phi _1) S_z I_{3x} \nonumber \\&+ B_4(\phi _2) S_z I_{4x} + B_5(\phi _2) S_z I_{5x} + B_6(\phi _2) S_z I_{6x} \ , \end{aligned}$$

where $\omega _S$ and $\omega _I$ are the electron and proton Zeeman frequency, respectively, and assignment of the hyperfine couplings to the rotor phases is listed in Table 1. The matrix representation of ${\mathcal {H}}_\mathrm {spin}$ has size $128 \times 128$. Each of the $R^2$ ro-librational states $(r_1, r_2)$ has 9 localized substates $(\phi _1, \phi _2)$. After including the spin degrees of freedom, each substate is represented by a $128 \times 128$ matrix, leading to dimension $1152 \times 1152$ for each ro-librational state. The total spin Hamiltonian ${\mathcal {H}}_\mathrm {spin}^\mathrm {total}$ is block-diagonal in this matrix representation and identical in all ro-librational states. The complete Hamiltonian for the uncoupled pair is thus given by

14 $$\begin{aligned} {\mathcal {H}}^\mathrm {loc} = {\mathcal {H}}_\mathrm {rot}^\mathrm {loc} \otimes {\mathbb {E}}_{128} + {\mathbb {E}}_{(2K+1)^2/9} \otimes {\mathcal {H}}_\mathrm {spin}^\mathrm {total} \ . \end{aligned}$$

For $K = 25$, corresponding to $R = 17$, matrix dimension of these operators is $332'928 \times 332'928$. Hence, a full treatment is not tenable, at least not on a desktop computer, and further truncation is required. We will address this problem after introducing the rotor-coupling term.

## Rotor-Rotor Coupling

In a simple approximation, rotor-rotor coupling is described by a Hamiltonian operator term [9]

15 $$\begin{aligned} {\mathcal {H}}_\mathrm {rrc} = W_3 \cos \left[ 3 \left( \phi _1 - \phi _2 \right) \right] \end{aligned}$$

with truncated matrix representation

16 $$\begin{aligned} {\mathcal {H}}_\mathrm {rrc} \approx \frac{W_3}{2} \left( {\mathbb {I}}_{-3} \otimes {\mathbb {I}}_{+3} + {\mathbb {I}}_{+3} \otimes {\mathbb {I}}_{-3} \right) \ , \end{aligned}$$

which at this point has dimension $\left( 2K+1\right) ^2 \times \left( 2K+1\right) ^2$. Equation (17) is determined by symmetry of the problem, except for a possible phase shift $\phi _0$ of the coupling term with respect to the phase of the first rotor. The general form

17 $$\begin{aligned} {\mathcal {H}}_\mathrm {rrc,gen} = W_3 \cos \left[ 3 \left( \phi _1 - \phi _2 \right) + \phi _0 \right] \end{aligned}$$

applies in all cases where interaction of the two methyl groups with the environment does not break symmetry between the groups. In analogy to the derivation in the Appendix of [9], the truncated Hamiltonian for the general case can be expressed as

18 $$\begin{aligned} {\mathcal {H}}_\mathrm {rrc,gen}&\approx \frac{W_3}{2}&\left[ \cos \phi _0 \left( {\mathbb {I}}_{-3} \otimes {\mathbb {I}}_{+3} + {\mathbb {I}}_{+3} \otimes {\mathbb {I}}_{-3} \right) \right.&\left. +i \sin \phi _0 \left( {\mathbb {I}}_{-3} \otimes {\mathbb {I}}_{+3} - {\mathbb {I}}_{+3} \otimes {\mathbb {I}}_{-3} \right) \right] \ . \end{aligned}$$

The following transformations work with the general form ${\mathcal {H}}_\mathrm {rrc,gen}$ as well as with ${\mathcal {H}}_\mathrm {rrc}$. Choice of this phase affects the value of $W_3$ at which a given tunnel frequency is obtained at a given value of $V_3$. We have checked that our general conclusions are not affected by this choice. In all example calculations, we therefore assume $\phi _0 = 0$.

The energy levels corresponding to ${\mathcal {H}}_\mathrm {rot}+{\mathcal {H}}_\mathrm {rrc}$, as defined in Eqs. (8) and (18), is shown in Fig. 1b. The term ${\mathcal {H}}_\mathrm {rrc}$ can be transformed to the localized basis in three steps. First, we transform ${\mathcal {H}}_\mathrm {rrc}$ to the eigenbasis of the rotor Hamiltonain for the two uncoupled rotors

19 $$\begin{aligned} {\mathcal {H}}_\mathrm {rrc}^{(1)} = \left( {{\varvec{V}}} \otimes {{\varvec{V}}} \right) ' {\mathcal {H}}_\mathrm {rrc} \left( {{\varvec{V}}} \otimes {{\varvec{V}}} \right) \end{aligned}$$

Second, we reorder states as described above for the uncoupled rotor Hamiltonian, using index expression Eq. (11). This provides the rearranged matrix representation ${\mathcal {H}}_\mathrm {rrc}^{(2)}$. Third, we transform to the localized basis

20 $$\begin{aligned} {\mathcal {H}}_\mathrm {rrc}^{(3)} = {{\varvec{L}}}' {\mathcal {H}}_\mathrm {rrc}^{(2)} {{\varvec{L}}} \ . \end{aligned}$$

**Fig. 3:**
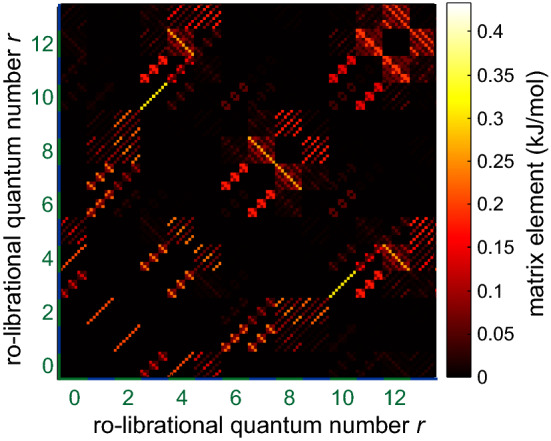
Matrix representation of the rotor-rotor coupling Hamiltonian ${\mathcal {H}}_\mathrm {rrc}^{(3)}$ in the localized basis for $V_3 = 8.785$ kJ/mol and $W_3 = -1$ kJ/mol. Only ro-librational levels $r = 0 \ldots 13$ are shown, corresponding to energies smaller than the rotational barrier

**Fig. 4:**
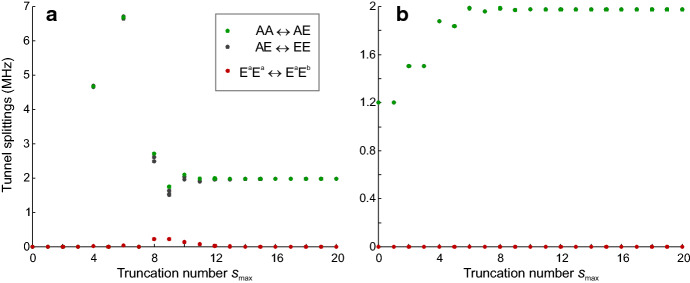
Convergence of tunnel splittings with truncation of the Hamiltonian matrix at increasing total ro-librational quantum number $s_\mathrm {max}$ in the regime $\left| W_3 \ll V_3 \right|$. Green dots correspond to AA$\leftrightarrow$AE transitions, grey dots to AE$\leftrightarrow$EE transitions and red dots indicate splitting between E$^\mathrm {a}$E$^\mathrm {a}$ and E$^\mathrm {a}$E$^\mathrm {b}$ levels. **a** $V_3 = 8.785$ kJ/mol and $W_3 = -1$ kJ/mol. Sufficient convergence is observed at $s_\mathrm {max} \ge 14$. **b** $V_3 = 12.313$ kJ/mol and $W_3 = 1$ kJ/mol. Sufficient convergence is observed at $s_\mathrm {max} \ge 10$ (Color figure online)

It is instructive to look at the off-diagonal matrix elements of ${\mathcal {H}}_\mathrm {rrc}^{(3)}$ that are of the order of $W_3$ (Fig. 3). In general, the coupling term mixes all $(2K+1)^2$ states to a substantial extent, well beyond the states visualized in Fig. 3. This suggests that further truncation may be problematic. However, as we shall see below, we are often interested in only the ro-librational ground state. The coupling term admixes excited ro-librational states to this ground state. As long as $W_3 \ll V_3$, energies and eigenvectors of the 9 substates of the ro-librational ground state are expected to converge for a low number of excitations as demonstrated in Fig. 4. For this analysis, we denote the excitation order by the maximum quantum number $s_\mathrm {max}$. As the number of ro-librational states for given $s = 0, 1, \ldots R-1$ equals $s+1$, the total number of ro-librational states to be considered up to $s_\mathrm {max}$ is $\left( s_\mathrm {max}+1\right) \left( s_\mathrm {max}+2\right) /2$. Hence, Hilbert space dimension $n_\mathrm {Hilbert}$ including spin degrees of freedom is

21 $$\begin{aligned} n_\mathrm {Hilbert} = \frac{9 \cdot 128 \cdot \left( s_\mathrm {max}+1\right) \left( s_\mathrm {max}+2\right) }{2} \end{aligned}$$

At $s_\mathrm {max} \ge 8$, matrix size becomes too large for full diagonalization on a desktop computer. Fortunately, the matrix representation of the Hamiltonian is sparse. Furthermore, for high rotation barriers, only the ro-librational ground state is significantly populated at temperatures of interest. Therefore, it suffices to compute the $9\cdot 128$ lowest eigenvalues and corresponding eigenvectors. In order to further reduce memory and computation time requirements in solving the coupled-rotor problem, we truncate ${\mathcal {H}}_\mathrm {rot}^\mathrm {loc}$ and ${\mathcal {H}}_\mathrm {rrc}^{(3)}$ to the $n_\mathrm {Hilbert}$ lowest levels at a value $s_\mathrm {max}$ where the tunnel frequencies are sufficiently converged. We thus obtain ${\mathcal {H}}_\mathrm {rot}^\mathrm {loc,trunc}$ and ${\mathcal {H}}_\mathrm {rrc}^{(3),\mathrm {trunc}}$. The Hamiltonian for the coupled-rotor problem including spin is then given by

22 $$\begin{aligned} {\mathcal {H}} = {\mathcal {H}}_\mathrm {rot}^\mathrm {loc,trunc} \otimes {\mathbb {E}}_{128} + {\mathcal {H}}_\mathrm {rrc}^{(3),\mathrm {trunc}} \otimes {\mathbb {E}}_{128} + {\mathbb {E}}_{n_\mathrm {Hilbert}/9} \otimes {\mathcal {H}}_\mathrm {spin}^\mathrm {total} \ . \end{aligned}$$

The effect of truncation at $s_\mathrm {max}$ on tunnel splittings can be studied without including spin states. This is possible because energy contributions by spin interactions are much smaller than splittings between ro-librational levels. As shown by Khazaei and Sebastiani [9], coupling only partially lifts degeneracy of the nine tunnel-split states of two methyl rotors. In the uncoupled case, the four AE states are degenerate and the four EE states are degenerate, as indicated in Fig. 1b. In the regime $W_3 \ll V_3$ that is of interest here, the tunnel splittings converge at values $s_\mathrm {max}$, where sub-space diagonalization up to the 1152 lowest eigenvalues is still feasible on a desktop computer. The convergence behavior for $V_3 = 8.785$ kJ/mol and $W_3 = -1$ kJ/mol is shown in Fig. 4a. At $s_\mathrm {max} \ge 14$, the three large splittings between the AA level and AE levels and between AE and EE levels coincide within resolution of ESEEM experiments and the splitting between E$^\mathrm {a}$E$^\mathrm {a}$ and E$^\mathrm {a}$E$^\mathrm {b}$ levels approaches zero. For $V_3 = 12.313$ kJ/mol and $W_3 = 1$ kJ/mol, such convergence is observed already for $s_\mathrm {max} \ge 10$ (Fig. 4b). We generally observe faster convergence at lower ratios of $\left| W_3/V_3 \right|$.

To set up quantum dynamics computations, we perform sub-space diagonalization of the Hamiltonian given by Eq. (22) for the $9\cdot 128 \times n_\mathrm {Hilbert}$ matrix ${\mathbf {W}}$ of eigenvectors, so that ${\mathcal {H}}_0^\mathrm {EB} = {{\varvec{W}}}' {\mathcal {H}} {{\varvec{W}}}$ is a $9\cdot 128 \times 9\cdot 128$ diagonal matrix that corresponds to the Hamiltonian of the tunnel and spin states in the lowest ro-librational state.

## Density Operator Computations

By density operator formalism, we can compute the signal for any pulse sequence applied to the coupled two-rotor-spin system. The thermal equilibrium density operator is given by

23 $$\begin{aligned} \rho _\mathrm {eq}^\mathrm {EB} = \frac{e^{-\hbar {\mathcal {H}}_0^\mathrm {EB}/ k_\mathrm {B} T} }{\mathrm {Trace}\left( e^{-\hbar {\mathcal {H}}_0^\mathrm {EB}/ k_\mathrm {B} T} \right) } \ , \end{aligned}$$

where $k_\mathrm {B}$ is Boltzmann’s constant and *T* the temperature. Spin operators $F_\xi$ ($F = S, I$, $\xi = x, y , z$) required for excitation or detection are expanded into the localized basis by

24 $$\begin{aligned} F_{\xi }^\mathrm {loc} = {\mathbb {E}}_{(2K+1)^2} \otimes F_\xi \ . \end{aligned}$$

Their representation in the eigenbasis of the Hamiltonian of the tunnel and spin states in the lowest ro-librational state is given by

25 $$F_{\xi }^{{{\text{EB}}}} = W^{\prime } {\mathcal{F}}_{\xi }^{{{\text{loc}}}} W.$$

Due to the Hilbert space dimension of 1152, such density operator computations are computationally expensive, but they are feasible.

## Separability of Barrier Height and Rotor-Rotor Coupling

We now test whether tunnel ESEEM can separate the rotation barrier $V_3$ and the rotor-rotor coupling $W_3$ in a regime expected for two methyl groups bound to the same sp$^3$ atom. To this end, we assume the model Hamiltonian defined by Eqs. (6) and (17). We restrict ourselves to the parameter range for $V_3$ and $W_3$ where tunnel ESEEM should be easily observable. This requires tunnel frequencies of the order of the difference between the hyperfine couplings to protons in the same methyl group, roughly between 100 kHz and 100 MHz, corresponding to $V_3$ between 15 and 5.8 kJ/mol for the uncoupled case. In particular, we assume a tunnel frequency $\nu _{\mathrm {t},0} = 1.9753$ MHz, corresponding to $V_3 = 10.5$ kJ/mol (1263 K). The suspicion that the two parameters might be unseparable arises from the fact that, at $W_3 \ll V_3$, the coupling splits the two E levels of each rotor only by a frequency difference that is smaller than the ESEEM linewidth. This indicates that the eigenfunctions are very close to products of the single-rotor eigenfunctions. If so, any pair of ($V_3$, $W_3$) that leads to the same $\nu _{\mathrm {t},0}$ will provide ESEEM data that are indistinguishable from each other by frequencies and modulation depth.

**Fig. 5:**
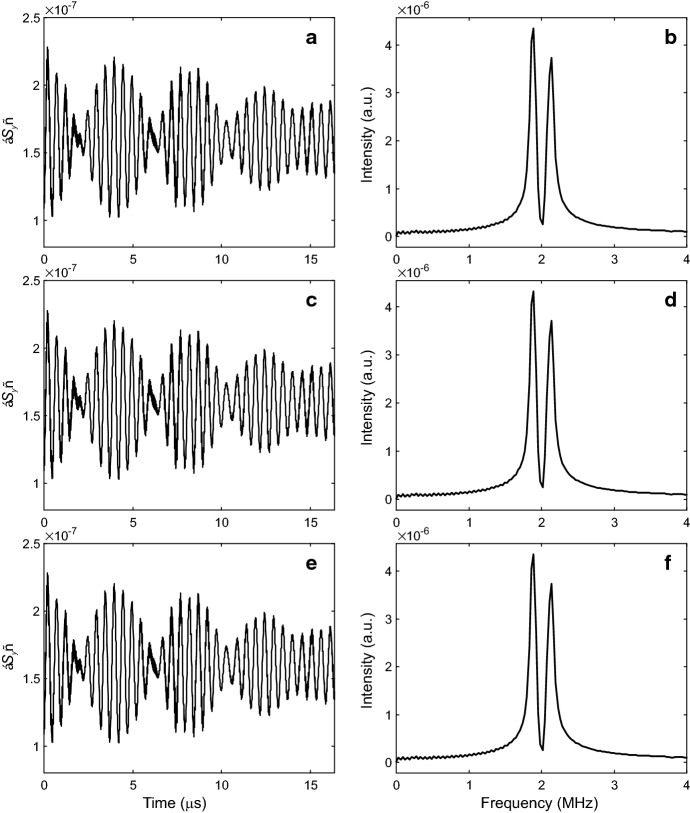
Simulated time-domain Q-band three-pulse tunnel ESEEM data at 10 K (**a**, **c**, **e**) and corresponding spectra in the tunnel frequency range (**b**, **d**, **f**) corresponding to different parameter pairs ($V_3$, $W_3$). Hyperfine couplings correspond to the geometry of the Mn(II)-DMA system shown in Fig. 2, assuming the single orientation where contributions of the two methyl groups to modulation are most similar in magnitude. The low-amplitude fast modulations in panels **a**, **c**, **e** correspond to nuclear frequencies near 50 MHz (not shown in **b**, **d**, **f**). **a**, **b** $V_3 = 10.5$ kJ/mol, $W_3 = 0$. **c**, **d** $V_3 = 8.785$ kJ/mol and $W_3 = -1$ kJ/mol. **e**, **f** $V_3 = 12.313$ kJ/mol for $W_3 = 1$ kJ/mol

In a first step, we considered the problem of two coupled rotors in the absence of electron and nuclear spin and assume $\left| W_3 \right| = 1$ kJ/mol. By variation of $V_3$ we find that $\nu _{\mathrm {t},0}$ is matched at $V_3 = 8.785$ kJ/mol for $W_3 = -1$ kJ/mol ($\nu _{\mathrm {t},0} = 1.9758$ MHz) and at $V_3 = 12.313$ kJ/mol for $W_3 = 1$ kJ/mol ($\nu _{\mathrm {t},0} = 1.9751$ MHz). In a second step, we compute ESEEM time-domain data and spectra for the parameter pairs $(V_3,W_3) = (10.5, 0), (8.785,-1), (12.313,1)$ kJ/mol using the approach described above. To that end, we assume hyperfine couplings as in Mn-doped [(CH$_3$)$_2$NH$_2$][Zn(HCOO)$_3$] [8] and a single orientation, where hyperfine splitting of the tunnel ESEEM peak is nicely resolved.

That way we simulated ESEEM data at a magnetic field of 1.175 T corresponding to Q-band measurements and a temperature of 10 K for all three parameter pairs (Fig. 5) with experimental parameters similar to the ones in our previous experimental work [8]. In particular, we assumed $\pi /2$ pulses of 10 ns length, a first interpulse delay $\tau = 148$ ns, a starting value $t_0 = 25$ ns for the second interpulse delay and 2048 data points with time increment of 8 ns. We apodized the data by a Dolph-Chebyshev window and zero-filled it to 4096 data points before Fourier transformation. This procedure corresponds to a best-case scenario for recognizing differences in the ESEEM data; in reality resolution will be lower and noise will be present. Further, by considering only a single orientation, we avoid anisotropic broadening present in powder ESEEM spectra. Despite all that, the time-domain data (Fig. 5a, c, e) and spectra (Fig. 5b, d, f) are identical within the remaining uncertainty due to an imperfect match of the tunnel frequency and due to truncation of the Hamiltonian. The differences in the time-domain data are less than 1% of the maximum amplitude of the echo modulation for the uncoupled case. We conclude that, at least for the Hamiltonian that we assumed here, tunnel ESEEM cannot separate barrier height $V_3$ and rotor-rotor coupling $W_3$ in the regime of small rotor-rotor coupling, where degeneracy of the EE states persists within ESEEM resolution.

**Fig. 6:**
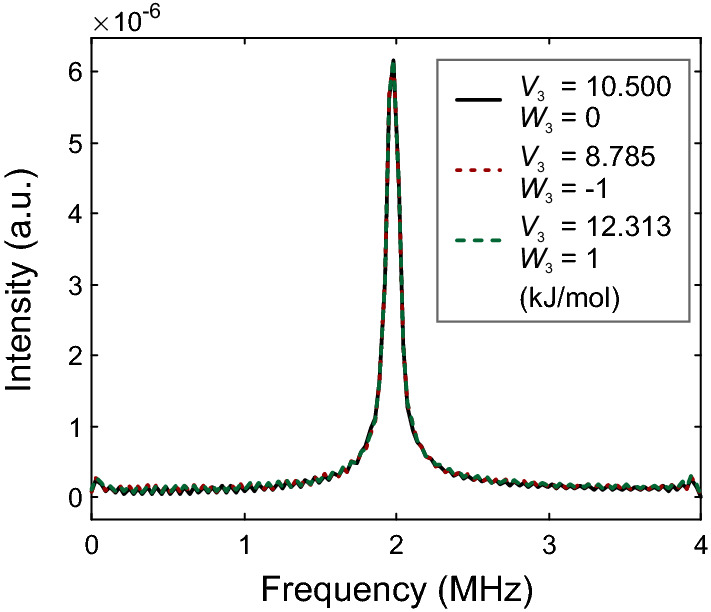
Simulated time-domain Q-band hyperfine-decoupled tunnel ESEEM spectra at 10 K corresponding to different parameter pairs ($V_3$, $W_3$). Only the tunnel frequency range is shown. Hyperfine couplings correspond to the geometry of the Mn(II)-DMA system shown in Fig. 2, assuming the single orientation where contributions of the two methyl groups to modulation are most similar in magnitude. The spectra correspond to $V_3 = 10.5$ kJ/mol, $W_3 = 0$ (black solid line), $V_3 = 8.785$ kJ/mol and $W_3 = -1$ kJ/mol (red dotted line), and $V_3 = 12.313$ kJ/mol for $W_3 = 1$ kJ/mol (green dashed line) (Color figure online)

We have then tested whether the same inseparability of $V_3$ and $W_3$ applies to hyperfine-decoupled tunnel ESEEM. This does not require recomputation of the Hamiltonian, equilibrium density operator, and spin operators for the ro-librational ground state. We assumed the same $\pi /2$ pulse length, the same microwave power for the high-turning angle pulse as for the $\pi /2$ pulse, and a minimum length of the HTA pulse of 20 ns, corresponding to a $\pi$ pulse. We simulated 2048 data points with a pulse length increment of 8 ns. Fig. 6 shows that, first, hyperfine decoupling of the tunnel ESEEM frequencies is expected to work under these conditions, and, second, the spectra for the three parameter sets coincide within the resolution that can be expected in such experiments. We obtained similar results for a different Mn(II) position and orientation, where only one of the two methyl groups exhibits significant modulation (not shown).

## Discussion

Our findings indicate that the multiple tunnel ESEEM peaks observed in Mn-doped [(CH$_3$)$_2$NH$_2$][Zn(HCOO)$_3$] and [(CH$_3$)$_2$NH$_2$][Cd(N)$_3$] under hyperfine decoupling [8] do not arise from rotational coupling between the two methyl groups of the DMA cation, as we had originally speculated. This conclusion is subject to a few caveats. First, we considered only relatively weak rotor-rotor coupling, as we had originally estimated from fitting relaxed potential surface scans of an isolated DMA cation at Kohn-Sham level. This is a minor limitation, since much larger couplings $W_3$ would require unrealistically small (negative $W_3$) or large (positive $W_3$) rotational barriers $V_3$ for a single methyl group. We did additionally test the parameter set $V_3 = 15$ kJ/mol, $W_3 = 2.41$ kJ/mol at $s_\mathrm {max} = 14$ and confirmed the same behavior as shown above for $\left| W_3 \right| = 1$ kJ/mol. Second, the simple coupling Hamiltonian specified by Eq. (18) only approximates rotor-rotor coupling of geminal methyl groups. This problem can only be solved by more intricate quantum dynamics computations. We shall address this in a collaborative effort. Third, our approach does not explicitly consider that nuclear spin states and tunnel states are symmetry related and that nuclear spin states must adhere to the Pauli principle. We do not think that this is a deficiency. The signals that we observe arise exclusively from electron spin thermal equilibrium polarization as an initial state. The experiments are simulated in density matrix formalism by Liouville-von-Neumann evolution under the proper system Hamiltonian, which should take account of any symmetry conditions that exist.

If our conclusion holds, the appearance of multiple tunnel frequencies under hyperfine decoupling may indicate heterogeneity of the metal-organic framework that is sensed by the methyl quantum rotors. A trivial source of such heterogeneity is manganese doping in itself. In the crystal structure of [(CH$_3$)$_2$NH$_2$] [Mn(HCOO)$_3$], which is assumed to be isomorphous to the one of [(CH$_3$)$_2$NH$_2$] [Zn(HCOO)$_3$], eight Mn(II) positions are sufficiently close to the DMA cation to induce tunnel ESEEM. By its orientation, the DMA cation breaks symmetry of this manganese cube, so that all eight Mn-DMA pairs are inequivalent. The two extreme Mn(II) positions with respect to the DMA cation are shown in Fig. 2. For the position marked Mn, the nitrogen atom of the DMA cation is at a distance of 4.440 Å from the Mn(II) ion, whereas for the position marked Mn’, it is at a distance of 5.936 Å. For the former position, the carbon atoms of the two methyl groups are at rather similar distances of 5.003 and 5.775 Å from the Mn(II) ion, whereas for the latter position they are at quite different distances of 4.598 and 7.053 Å. The ionic radii of Mn(II) (0.97 Å) and Zn(II) (0.88 Å) in octahedral coordination differ and so do bond order sums computed from coordination bond lengths [12]. It appears feasible that, depending on the DMA neighbor site occupied by Mn(II) in an otherwise Zn(II) framework, the cage is deformed in a different way, which might even lead to a reorientation of the DMA cation. Since tunnel splitting is exponentially sensitive to the height of the methyl rotation barrier, this effect might cause the distribution of tunnel frequencies observed in these systems.

Alternatively, the additional peaks might be a result of the group spin $S = 5/2$ of Mn(II). In fact, hyperfine decoupling in electron-nuclear ESEEM is known to be incomplete for high-spin systems [13]. Preliminary simulations for a single orientation indicate that the same applies to tunnel ESEEM. However, full powder simulations of this effect are beyond the scope of the current paper and a safe conclusion could only be drawn if such simulations would reproduce the additional peaks. In any case, the preliminary results suggest to study the effect of hyperfine decoupling on tunnel ESEEM on an $S=1/2$ system.

Further, if our conclusion holds that weak rotational coupling is unseparable by ESEEM experiments from a change in the rotation barrier, potential effects of methyl tunneling on electron spin decoherence [14] can be studied with the 48-state model introduced in [8] also for the geminal methyl groups in nitroxide spin labels. Thus, the 1152-state model introduced here would not be needed for this case, leading to substantial savings in computational expense. However, a caveat exists here, too, if we consider general treatment of methyl-tunneling-induced decoherence. For the much lower rotation barriers encountered for methyl groups bound to sp$^2$ hybridized second-row elements [15], the $\left| W_3/V_3 \right|$ ratio is likely to fall outside the weak-coupling regime that we have treated here. In fact, for crystalline 4-methyl pyridine this has been demonstrated, both, by experiment [16, 17] and computation [9]. Hence, for solvents such as toluene or functional groups such as acetyl groups, a proper treatment may become even more complicated than the approach followed here. In the glassy state typical for samples in EPR application work, one might then expect a broad distribution of rotation barriers and rotor-rotor couplings, corresponding to an extremely broad distribution of tunnel splittings. In fact, the concept of individual tunnel splitting of a single methyl group may then break down.

## Conclusion

For rotor-rotor couplings much smaller than the rotation barrier, the 1192-quantum state problem of two tunnel-split methyl groups in the vicinity of an electron spin can be solved with moderate computational effort. The numerical solution indicates that, in this regime, the coupling changes only the tunnel splitting compared to the case with the same rotation barrier in the absence of coupling. It does not lead to a qualitative difference in the hyperfine-mediated tunnel ESEEM effect. As a consequence, in this regime ESEEM data depend only on the tunnel splitting and not on the particular combination of single-rotor potential and rotor-rotor coupling that causes this splitting. This applies also to hyperfine-decoupled ESEEM, where the pure tunnel splitting is recovered. If these conclusions hold also for a more sophisticated treatment of the coupled-methyl rotor tunneling problem, electron spin decoherence induced by methyl groups bound to sp$^{3}$ hybridized atoms may find a simple description. In contrast, the case of methyl groups bound to sp$^{2}$ hybridized atoms may require either an improved treatment or substantial computational resources. We plan further experimental and theoretical work along these lines.
